# Isolated Right Ventricular Myocarditis: Rarely Reported Pathology

**DOI:** 10.1155/2015/790246

**Published:** 2015-09-02

**Authors:** Hafeez Ul Hassan Virk, Muhammad Bilal Munir

**Affiliations:** ^1^Department of Internal Medicine, Mount Sinai Icahn School of Medicine, St. Luke's Roosevelt Hospital, New York, NY 10027, USA; ^2^Department of Internal Medicine, UPMC Montefiore/Presbyterian Hospital, University of Pittsburgh Medical Center, Pittsburgh, PA 15213, USA

## Abstract

*Objective.* Preventing the morbidity and mortality from isolated right ventricular myocarditis by its early recognition and treatment. *Background*. The clinical presentation of myocarditis ranges from nonspecific systemic symptoms (fever, myalgia, palpitations, or exertional dyspnea) to fulminant cardiac failure and sudden death. In our case, echocardiography raised the possibility of myocarditis at an early stage, although the signs and symptoms did not indicate right ventricular disease. Review of the literature showed only 4 previous reports, all diagnosed at autopsy, in which diagnosis was not suspected in vivo. *Design/Methods*. We are reporting case of a 23-year-old male with no past medical history who presented to emergency room with a nonexertional sharp left sided chest pain. Diagnostic tests were conducted, which revealed elevated troponins, decreased right ventricular ejection function but preserved left ventricular function, and no evidence of coronary artery disease. *Results*. A diagnosis of isolated right ventricular myocarditis was made on the basis of clinical, echocardiographic, and cardiac MRI findings. *Conclusions*. Isolated right ventricular myocarditis should be suspected in a patient with depressed right ventricular function without left ventricular involvement on echocardiography and cardiac MRI, elevated cardiac enzymes, and no evidence of coronary artery disease.

## 1. Introduction

Myocarditis is the “inflammation of the myocardium.” Conventionally, Dallas criteria which are used to define the myocarditis require inflammatory cellular infiltrates on stained heart tissue sections [1]. These infiltrates may or may not damage the myocytes. Considering the risks of biopsy, some studies suggest cardiac MRI as an alternate method for diagnosis as abnormal signs on cardiac MRI correlate with regions of myocarditis [2, 3].

Its clinical presentation ranges from nonspecific systemic symptoms (fever, myalgia, palpitations, or exertional dyspnea) to fulminant cardiac failure and sudden death [4]. Acute myocarditis may cause substantial myocardial damage and can lead to arrhythmias, sudden cardiac death in the acute phase, and dilated cardiomyopathy in the acute and chronic phase. If not fatal, these complications can lead to chronic heart failure in the long term. Therefore, early diagnosis is of particular clinical importance and endomyocardial biopsy is the gold standard for diagnosis [4–6].

Nonspecific myocarditis can be seen in a variety of conditions which include viral diseases, drug induced hypersensitivity syndromes, and autoimmune diseases (giant cell myocarditis and cardiac sarcoidosis), but myocarditis unrelated to any other cause (isolated, idiopathic, or Fiedler's myocarditis) is occasionally found at autopsy [7]. Myocarditis (nonspecific or idiopathic) is mostly assumed to involve both ventricles equally or less commonly the left ventricular alone. Isolated involvement of right ventricle has been described in 4 case reports which may be either because of its rare incidence or because of underreporting due to lack of sufficient diagnostic guidelines and cases reported [8–10]. And only one of such reported cases survived because of early diagnosis and treatment [9].

## 2. Case Presentation

We are reporting case of a 23-year-old male with no past medical history who presented to emergency room with a nonexertional sharp left sided chest pain. The chest pain was preceded by two days of nonspecific flu-like symptoms (fatigue, cough, sinus congestion, and rhinorrhea). He denied any close sick contact, recent travel, or recent hospitalization. There was no family history of heart disease as well.

On examination, the patient appeared ill but was alert and oriented to person, place, and time. The mucous membranes were dry. Cardiac examination revealed regular tachycardia without extra heart sounds. There was no jugular venous distention. The lungs, abdomen, and skin were normal. Vitals were as follows: temperature: 38.7°C, pulse: 116, R/R: 18, B.P: 99/52, and pulse oximetry: 100%. Initial labs were normal with the exception of mild elevation of WBCs and initial troponins of 6.464 (reference range: 0–0.034 ng/mL) which trended up to 197.00.

**Figure 1:**
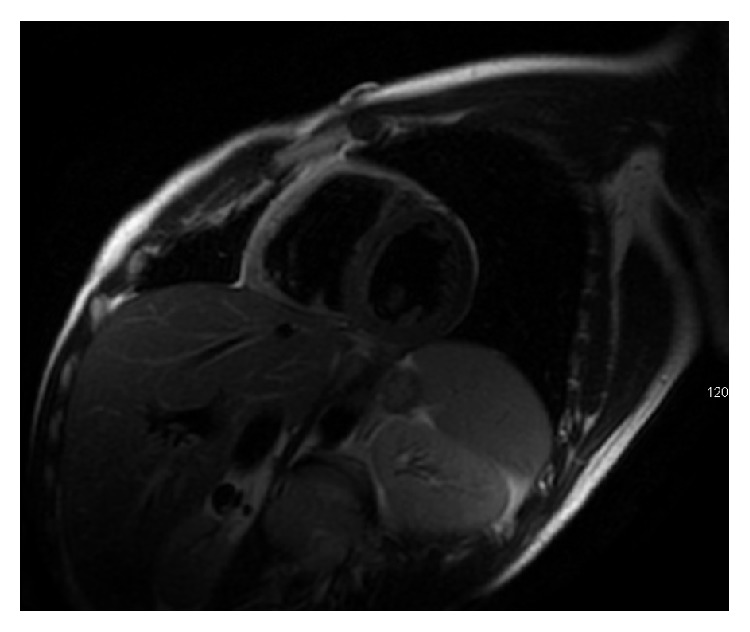
T2 weighted image showing diffuse RV edema.

**Figure 2:**
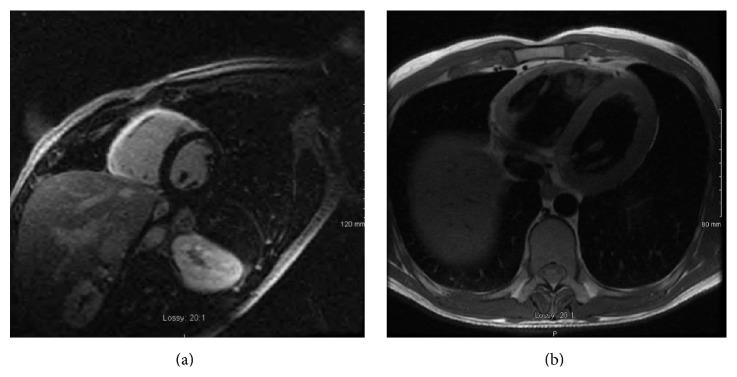
(a) Hyperemia on T1 weighted after gadolinium. (b) Fibrosis on transmural late gadolinium enhancement.

Chest radiograph revealed no air way disease or cardiac enlargement. The EKG showed sinus tachycardia with no ST or T wave changes and early echocardiogram revealed mild right ventricle hypokinesis. The right and left ventricular ejection fractions were 40% and 60%, respectively, with no valvular abnormality. Urgent cardiac catheterization was done which did not show any coronary artery disease. Cardiac MRI was done which showed diffuse edema on T2 weighted image (Figure 1), hyperemia on T1 weighted after gadolinium (Figure 2(a)), and fibrosis on transmural late gadolinium enhancement (Figure 2(b)) of the wall of the right ventricle, consistent with isolated RV myocarditis. RV ejection fraction (EF) was found to be 44% (normal: male = 47–74%, female = 47–80%) while RV end diastolic (RVEDV) and end systolic volumes (RVESV) were 232.5 mL and 129.2 mL, respectively (RVEDV: normal: male = 88–227 mL, female = 58–154 mL and RVESV: normal: male = 23–105 mL, female = 12–68 mL). Troponin level started trending down next day and leukocytosis resolved.

His blood cultures were negative and complete work-up of the patient failed to reveal any specific cause of myocarditis. His respiratory panel was negative for viral pathogens as cause of respiratory illness leading to isolated right sided myocarditis. He was discharged with high dose nonsteroidal anti-inflammatory agents. His repeated MRI after 8 weeks showed resolution of the RV wall edema and improvement in ventricular ejection fraction.

## 3. Discussion

Nonspecific myocarditis can be seen in a variety of conditions including hepatitis C infection, HIV, autoimmune diseases, rheumatoid diseases, certain hematologic abnormalities, medications, and small pox vaccination [4, 5]. However, myocarditis can be unrelated to any other disease process and is occasionally found only at autopsy. This type of myocarditis has been called isolated, idiopathic, or Fiedler's myocarditis [7] and can be a nonspecific response to triggers such as viral or bacterial infection, cardiotoxic agents, catecholamines, infarction, or mechanical injury [9].

Myocarditis should be suspected in patients who present, with or without signs of compromised cardiac function, with a rise in cardiac biomarker levels, ECG findings suggestive of acute myocardial injury, new onset arrhythmia, or abnormalities of ventricular systolic function, particularly if such clinical findings are new and other common causes are excluded [4, 8, 11].

Both nonspecific and isolated myocarditis are assumed to involve both ventricles diffusely or less commonly the left ventricle alone. Right ventricular involvement alone has been rarely reported [8].

Isolated right ventricular myocarditis was first described by Hayes in 1961 and later by Mancio et al. in 2013 [9, 10]. Only four reported cases of isolated right ventricular myocarditis could be found from the literature search which could be because of the rare incidence of this disease or because of underreporting of less serious cases of isolated right ventricular myocarditis as it lacks sufficient diagnostic and treatment guidelines.

Myocarditis may present with symptoms ranging from chest pain to new-onset heart failure, atrial or ventricular arrhythmias, cardiogenic shock, and sudden death. Chronic heart failure from dilated cardiomyopathy is the major long-term sequel of myocarditis [5, 9]. In the case reported by Hayes, the patient did not manifest any of the signs and symptoms of myocarditis and lack of initiation of early treatment led to sudden death of patient. Isolated right ventricular myocarditis was later diagnosed on autopsy. However, in the case reported by Mancio and in our case, the patient presented with chest pain which was preceded by flu-like illness. Diagnostic tests were conducted in picture of clinical suspicion including cardiac enzymes, echocardiography, cardiac MRI, and cardiac catheterization which revealed elevated troponins, decreased right ventricular ejection function but preserved left ventricular function, and no evidence of coronary artery disease.

With early diagnosis and treatment with high dose NSAIDs, the patient's condition and right ventricular function improved although complete work-up of the patient failed to reveal any specific cause of myocarditis. Heart failure medications were not started as patient did not have any signs and symptoms of heart failure. Recently, NSAIDs have shown to be ineffective in animal models [12, 13] and are actually avoided in heart failure population as they lead to heart failure exacerbation and increase mortality. Endomyocardial biopsy was not considered as cardiac MRI results and response to therapy confirmed the diagnosis [5, 9].

## 4. Conclusion

Isolated right ventricular myocarditis should be suspected in a patient with depressed right ventricular function without left ventricular involvement on echocardiography and cardiac MRI, elevated cardiac enzymes with clean coronary arteries.

Early diagnosis and treatment should be prompted to improve right ventricular function and to prevent progression to a more serious sequel and death. Heart failure medications should be started if patient is with heart failure secondary to myocarditis. We encourage other centers to report similar cases so that definitive diagnosis and treatment guidelines could be established for this rare disease which if ignored can cause early death.
